# Avelumab, an anti-PD-L1 antibody, in patients with locally advanced or metastatic breast cancer: a phase 1b JAVELIN Solid Tumor study

**DOI:** 10.1007/s10549-017-4537-5

**Published:** 2017-10-23

**Authors:** Luc Y. Dirix, Istvan Takacs, Guy Jerusalem, Petros Nikolinakos, Hendrik-Tobias Arkenau, Andres Forero-Torres, Ralph Boccia, Marc E. Lippman, Robert Somer, Martin Smakal, Leisha A. Emens, Borys Hrinczenko, William Edenfield, Jayne Gurtler, Anja von Heydebreck, Hans Juergen Grote, Kevin Chin, Erika P. Hamilton

**Affiliations:** 1 Sint Augustinus-University of Antwerp, Antwerp, Belgium; 2 Semmelweis University, Budapest, Hungary; 3 CHU Sart Tilman Liege and Liege University, Liege, Belgium; 4 University Cancer & Blood Center, LLC, Athens, GA USA; 5 Sarah Cannon Research Institute, London, UK; 6 University College London Cancer Institute, London, UK; 7 University of Alabama, Birmingham, AL USA; 8 Center for Cancer and Blood Disorders, Bethesda, MD USA; 9 University of Miami Miller School of Medicine, Miami, FL USA; 10 Cooper Hospital University Medical Center, Camden, NJ USA; 11 Nemocnice Horovice, Onkologicke Oddelení, Horovice, Czech Republic; 12 The John Hopkins University School of Medicine, Baltimore, MD USA; 13 Michigan State University, East Lansing, MI USA; 14 Greenville Hospital System, Greenville, SC USA; 15 Metairie Oncologist LLC, Metairie, LA USA; 16 Merck KGaA, Darmstadt, Germany; 17 EMD Serono, Billerica, MA USA; 18 Sarah Cannon Research Institute, Nashville, TN USA

**Keywords:** Avelumab, Metastatic breast cancer, Triple-negative breast cancer, PD-L1, Second-line

## Abstract

**Purpose:**

Agents targeting programmed death receptor 1 (PD-1) or its ligand (PD-L1) have shown antitumor activity in the treatment of metastatic breast cancer (MBC). The aim of this study was to assess the activity of avelumab, a PD-L1 inhibitor, in patients with MBC.

**Methods:**

In a phase 1 trial (JAVELIN Solid Tumor; NCT01772004), patients with MBC refractory to or progressing after standard-of-care therapy received avelumab intravenously 10 mg/kg every 2 weeks. Tumors were assessed every 6 weeks by RECIST v1.1. Adverse events (AEs) were graded by NCI-CTCAE v4.0. Membrane PD-L1 expression was assessed by immunohistochemistry (Dako PD-L1 IHC 73-10 pharmDx).

**Results:**

A total of 168 patients with MBC, including 58 patients with triple-negative breast cancer (TNBC), were treated with avelumab for 2–50 weeks and followed for 6–15 months. Patients were heavily pretreated with a median of three prior therapies for metastatic or locally advanced disease. Grade ≥ 3 treatment-related AEs occurred in 13.7% of patients, including two treatment-related deaths. The confirmed objective response rate (ORR) was 3.0% overall (one complete response and four partial responses) and 5.2% in patients with TNBC. A trend toward a higher ORR was seen in patients with PD-L1+ versus PD-L1− tumor-associated immune cells in the overall population (16.7% vs. 1.6%) and in the TNBC subgroup (22.2% vs. 2.6%).

**Conclusion:**

Avelumab showed an acceptable safety profile and clinical activity in a subset of patients with MBC. PD-L1 expression in tumor-associated immune cells may be associated with a higher probability of clinical response to avelumab in MBC.

## Introduction

Despite advances in the treatment of breast cancer and an encouraging 5-year overall survival rate of approximately 90% in the United States, up to 30% of patients with an early-stage diagnosis eventually progress to incurable metastatic disease, and 6% of patients have metastatic disease at diagnosis [1, 2]. Treatment of metastatic breast cancer (MBC) is based on molecular subtype and may include chemotherapy, human epidermal growth factor receptor 2 (HER2)-directed agents, and endocrine-based therapies or agents targeted to mechanistic target of rapamycin or CDK4/6 for those tumors overexpressing estrogen receptor (ER) and/or progesterone receptor (PR) [3]. Approximately, 15–20% of patients have breast cancers lacking expression of ER, PR, or HER2, which are termed triple-negative breast cancer (TNBC). For these patients, standard treatment is cytotoxic chemotherapy, which is limited by poor tolerability and short duration of response [4–6]. Thus, new therapies are needed for patients with MBC whose disease has progressed following standard therapies.

Immune checkpoint inhibitors, particularly agents targeting programmed death receptor 1 (PD-1) or its ligand (PD-L1), are being increasingly explored as a potential treatment strategy in various cancers [7]. Breast cancers express PD-L1, with higher expression often seen in TNBC tumors [8–10]. Binding of PD-L1 to its receptor on T cells, PD-1, inhibits adaptive immune responses in the tumor microenvironment, which enables tumor cell escape from immune cells [11–13]. The presence of tumor-infiltrating lymphocytes (TILs) in breast cancer has been shown to have a strong prognostic association [14]. High TIL levels are associated with tumors having PD-L1 expression, and PD-L1+ tumors with high TILs have better outcomes [9, 10, 15]. PD-L1 expression may serve as a marker of immune activity, and local immunosuppression of TILs via the PD-L1/PD-1 pathway may be an important means of tumor immune evasion [8, 14]. Inhibition of the PD-L1/PD-1 axis with monoclonal antibodies may be one means of restoring immune surveillance and cell-mediated antitumor activity, and studies of anti-PD-L1/PD-1 agents have shown durable antitumor responses in patients with various advanced cancers [7, 16]. Early-phase studies have suggested that these agents may also have clinical activity in breast cancer, particularly in the TNBC subtype [17–19].

Avelumab (MSB0010718C) is a human anti-PD-L1 IgG1 monoclonal antibody that inhibits the interaction between PD-1 and PD-L1, leaving PD-1/PD-L2 interactions intact [20]. Unlike other anti-PD-L1/PD-1 antibodies approved or in advanced clinical development, avelumab has been shown to induce antibody-dependent cell-mediated cytotoxicity (ADCC) of tumor cells in preclinical studies, suggesting it may potentially have an additional mechanism of action [21–23]. In phases 1 and 2 clinical studies, avelumab has been well tolerated and associated with durable responses in patients with various advanced tumors, including Merkel cell carcinoma (MCC), non-small cell lung cancer, and urothelial carcinoma [20, 24–26]. Avelumab is approved by the US Food and Drug Administration for the treatment of metastatic MCC and locally advanced or metastatic urothelial carcinoma that has progressed during or after platinum-containing chemotherapy [27]. In the phase 1a part of the JAVELIN Solid Tumor study, avelumab was safely administered by intravenous infusion every 2 weeks and had a predictable pharmacokinetic profile at doses of up to 20 mg/kg; the 10 mg/kg dose was selected for further study in phase 1b dose-expansion cohorts enrolling a range of tumor types [20]. Here, we report the evaluation of avelumab in a phase 1b cohort of patients with MBC as part of the JAVELIN Solid Tumor trial.

## Methods

### Study design and patients

JAVELIN Solid Tumor is an international, open-label, phase 1 trial in patients with advanced solid malignancies. In the dose-expansion cohort reported here, eligible patients had histologically confirmed locally advanced or MBC that was refractory to or had progressed after standard-of-care therapy. Eligible patients were aged ≥ 18 years and had an Eastern Cooperative Oncology Group (ECOG) performance status of 0 or 1, an estimated life expectancy of > 3 months, and adequate hepatic, renal, and hematologic function. Patients had received ≤ 3 prior lines of cytotoxic therapy (excluding systemic therapy that was not considered cytotoxic) and, unless contraindicated, were required to have received prior treatment with a taxane and anthracycline in any therapeutic setting. Patients had ≥ 1 measurable lesion per Response Evaluation Criteria In Solid Tumors (RECIST) version 1.1 [28]. A biopsy or surgical specimen for biomarker testing collected within 90 days prior to the first avelumab administration was required. Patients were unselected for PD-L1 expression and breast cancer subtype. Patients were enrolled in accordance with an approved protocol, international standards of good clinical practice, and institutional safety monitoring, and written informed consent was provided by patients.

### Procedures and assessments

Patients received avelumab (EMD Serono, Research & Development Institute, Billerica, MD, USA, a business of Merck KGaA, Darmstadt, Germany) 10 mg/kg intravenously every 2 weeks until confirmed disease progression, unacceptable toxicity, or other protocol-based criteria for withdrawal occurred. Safety and tolerability were assessed per the National Cancer Institute’s Common Terminology Criteria for Adverse Events (NCI-CTCAE), version 4.0. Potential immune-related adverse events (AEs) were identified using a prespecified list of Medical Dictionary for Regulatory Activities terms. Signs and symptoms of an infusion-related reaction, such as fever, chills, or rigors reported on the same day or the day following treatment, were queried with investigators to ascertain whether an AE of infusion-related reaction should be recorded. Premedication with diphenhydramine and acetaminophen was required 30–60 min before all infusions of avelumab to mitigate the occurrence of infusion-related reactions. Tumors were evaluated radiographically at baseline and every 6 weeks for the first 12 months, then every 12 weeks thereafter. Best overall response, duration of response, and progression-free survival were determined according to RECIST version 1.1 per investigator.

HER2, ER, and PR statuses were obtained from patient records. Levels of PD-L1 protein expressed on tumor cell membranes and on membranes and/or cytoplasm of immune cells within the tumor microenvironment were assessed by immunohistochemistry (IHC) staining of formalin-fixed, paraffin-embedded tissue sections of the most recent suitable biopsy or surgical specimen using a proprietary assay (PD-L1 IHC 73-10 pharmDx; Dako, Carpinteria, CA, USA) with an anti-PD-L1 rabbit monoclonal antibody. PD-L1 expression was assessed prospectively by central independent reviewers, who were blinded to any clinical data; expression was based on the percentages of tumor cells expressing PD-L1: 1 and 5% thresholds with any staining intensity and a 25% threshold with moderate to high staining. Additionally, dense aggregates of tumor-associated immune cells (identified as nonmalignant cells based on morphology) adjacent to tumor cells were assayed using a defined threshold of 10% of immune cells expressing PD-L1 at any staining intensity.

### Statistical methods

Enrollment of approximately 150 patients was planned for this cohort, and safety and activity were analyzed in all patients who received ≥ 1 dose of avelumab. The prespecified primary analysis occurred 6 months after the date of the first dose in the last patient enrolled. The objective response rate (ORR), defined as the proportion of patients with a confirmed best response of complete or partial response, was calculated with corresponding Clopper–Pearson CIs. Time-to-event endpoints (duration of response, progression-free survival, and overall survival) were estimated using Kaplan–Meier method, and CIs for the median were determined using the Brookmeyer–Crowley method. The trial is registered with ClinicalTrials.gov as NCT01772004.

## Results

### Patients

Of 266 patients screened, 168 patients with histologically confirmed MBC refractory to or progressing after standard-of-care therapy were enrolled and treated with avelumab between November 2013 and February 2015 (Table 1, Appendix Tables 4, 5). Of these 168 patients, 26 patients (15.5%) had HER2-positive disease (irrespective of ER and PR status), 72 patients (42.9%) had hormone-receptor-positive/HER2-negative disease, and 58 patients (34.5%) had TNBC. Median age was 55 (range 31–81) years. Patients had received a median of three prior therapies for metastatic disease, and 123 patients (73.2%) had received ≥ 2 prior anticancer regimens for metastatic or locally advanced disease (Table 1; Appendix Tables 4, 6). Median time since diagnosis of metastatic disease was 22 months (range 3 weeks to > 14.7 years). Of 58 patients with TNBC, 50% had received ≥ 2 prior lines of therapy for metastatic disease and median time since diagnosis was 13 months. Tumor specimens were evaluable for PD-L1 expression in 136 patients (81.0%), and based on a threshold of ≥ 1% tumor cell staining, 85 of 136 (62.5%) had PD-L1+ tumors. Using a ≥ 10% threshold for PD-L1 expression in tumor-associated immune cells, 12 of 136 evaluable patients (8.8%) had PD-L1+ tumors.

**Table 1:** Selected baseline characteristics

Characteristics	Total population *N* = 168	TNBC subgroup (*n* = 58)
Median age, years (range)	55 (31–81)	52.5 (31–80)
Age category, *n* (%) (years)		
< 65	140 (83.3)	54 (93.1)
≥ 65	28 (16.7)	4 (6.9)
Sex, *n* (%)		
Male	1 (0.6)	0
Female	167 (99.4)	58 (100)
Race or ethnic group, *n* (%)		
White	143 (85.1)	45 (7.8)
Black or African American	16 (9.5)	9 (15.5)
Asian	3 (1.8)	1 (1.7)
Other	6 (3.6)	3 (5.2)
Geographic region, *n* (%)		
United States	112 (66.7)	48 (82.8)
Europe	56 (33.3)	10 (17.2)
ECOG PS, *n* (%)		
0	83 (49.4)	33 (56.9)
1	85 (50.6)	25 (43.1)
Smoking history, *n* (%)		
Never smoker	107 (63.7)	36 (62.1)
Current or former smoker	50 (29.8)	17 (29.3)
Unknown	11 (6.5)	5 (8.6)
Histological subtype of tumor, *n* (%)		
Ductal	94 (56.0)	36 (62.1)
Lobular	6 (3.6)	0
Carcinoma, not otherwise specified	14 (8.3)	6 (10.3)
Other^a^	54 (32.1)	16 (27.6)
Molecular subtype, *n* (%)		
TNBC	58 (34.5)	58 (100)
HER2−/ER+ or PR+	72 (42.9)	–
HER2+	26 (15.5)	–
Unknown^b^	12 (7.1)	–
Median time since first diagnosis, months (range)	53.5 (7.3–407.5)	40.3 (7.3–241.0)
Median time since diagnosis of metastatic disease, months (range)^c^	21.6 (0.7–176.8)	13.2 (0.7–176.8)
Prior anticancer lines of therapy for metastatic or locally advanced disease, *n* (%)^d^		
≤ 1	45 (26.8)	29 (50.0)
2	35 (20.8)	16 (27.6)
≥ 3	88 (52.4)	13 (22.4)
Median (range)	3 (0–10)	2 (1–6)

PD-L1 expression status, *n*/*N* (%)^e^	PD-L1+	PD-L1−	PD-L1+	PD-L1−
≥ 1% tumor cells	85/136 (62.5)	51/136 (37.5)	33/48 (68.8)	15/48 (31.2)
≥ 5% tumor cells	23/136 (16.9)	113/136 (83.1)	13/48 (27.1)	35/48 (72.9)
≥ 25% tumor cells	3/136 (2.2)	133/136 (97.8)	2/48 (4.2)	46/48 (95.8)
≥ 10% tumor-associated immune cells	12/136 (8.8)	124/136 (91.2)	9/48 (18.8)	39/48 (81.2)

*ECOG PS* Eastern Cooperative Oncology Group performance status, *ER* estrogen receptor, *HER2* human epidermal growth factor receptor 2, *PD*-*L1* programmed death-ligand 1, *PR* progesterone receptor, *TNBC* triple-negative breast cancer

^a^Patients who were uncoded (overall, 41; TNBC, 11), other histology (overall, 10; TNBC, 5), or missing (overall, 3)

^b^Unknown molecular subtype was due to incomplete information in the medical records database (ER/PR status known, but HER2 status unknown in four patients) or to information collected retrospectively (molecular subtype status was from post-baseline samples in eight patients and therefore was not used for baseline characterization)

^c^Time since diagnosis of metastatic disease was missing for eight patients in the overall study population and six patients in the TNBC subgroup

^d^Regimen for metastatic disease may have included hormonal therapy, either alone or in combination with chemotherapy. Systemic therapies that were not necessarily cytotoxic are included in the number of prior regimens reported here, but the number of prior cytotoxic therapies permitted was ≤ 3

^e^Non-evaluable specimens included those that were missing, of poor quality or quantity (insufficient tissue on slide or insufficient tumor sample), or otherwise not available to provide results; all biopsy or surgical specimens were required to be collected within 90 days of first administration of avelumab

At the time of data cutoff (February 27, 2015), patients had received a median of four avelumab (range 1–23) doses and had a median treatment duration of 8.0 (range 2–50) weeks. Median duration of follow-up was 10.0 (range 6.0–15.2) months, and nine patients (5.4%) remained on avelumab treatment at data cutoff. Disease progression was the most common reason for treatment discontinuation (74.4%).

### Safety

Treatment-related AEs of any grade occurred in 115 patients (68.5%), including a grade ≥ 3 event in 23 patients (13.7%; Table 2; Appendix Table 7). The most commonly occurring treatment-related AEs of any grade (> 10% of patients) were fatigue (19.0%), infusion-related reaction (14.3%), and nausea (13.1%). Treatment-related AEs of any grade classified as immune-related occurred in 17 patients (10.1%): hypothyroidism (4.8%), autoimmune hepatitis and pneumonitis (1.8% each), thrombocytopenia (1.2%), and antinuclear antibody production, dry eye, elevated rheumatoid factor, hyperthyroidism, and pemphigoid skin reaction (0.6% each; Appendix Table 8). Four patients (2.4%) had a grade ≥ 3 immune-related, treatment-related AE (Appendix Table 8), including three patients (1.8%) with grade 3 autoimmune hepatitis and one patient each with grade 3 pneumonitis and grade 4 thrombocytopenia (0.6% each). One patient with autoimmune hepatitis who had progressive liver metastasis died of acute liver failure.

**Table 2:** Treatment-related adverse events occurring at any grade in ≥ 5% of patients or grade ≥ 3 in any patient

*N* = 168	Any grade	Grades 1–2	Grade 3	Grade 4	Grade 5
Any event, *n* (%)	115 (68.5)	92 (54.8)	16 (9.5)	5 (3.0)	2 (1.2)
Fatigue	32.9 (19.0)	29 (17.3)	3 (1.8)	0	0
Infusion-related reaction	24 (14.3)	24 (14.3)	0	0	0
Nausea	22 (13.1)	22 (13.1)	0	0	0
Diarrhea	15 (8.9)	15 (8.9)	0	0	0
Arthralgia	13 (7.7)	12 (7.1)	1 (0.6)	0	0
Decreased appetite	12 (7.1)	12 (7.1)	0	0	0
Influenza-like illness	11 (6.5)	11 (6.5)	0	0	0
Dyspnea exertional	5 (3.0)	4 (2.4)	1 (0.6)	0	0
Elevated AST	4 (2.4)	3 (1.8)	1 (0.6)	0	0
Elevated GGT	4 (2.4)	1 (0.6)	1 (0.6)	2 (1.2)	0
Anemia	3 (1.8)	0	2 (1.2)	1 (0.6)	0
Autoimmune hepatitis	3 (1.8)	0	3 (1.8)	0	0
Elevated ALT	3 (1.8)	2 (1.2)	1 (0.6)	0	0
Hypoxia	3 (1.8)	2 (1.2)	1 (0.6)	0	0
Pneumonitis	3 (1.8)	2 (1.2)	1 (0.6)	0	0
Axillary pain	2 (1.2)	1 (0.6)	1 (0.6)	0	0
Thrombocytopenia	2 (1.2)	1 (0.6)	0	1 (0.6)	0
Acute hepatic failure	1 (0.6)	0	0	0	1 (0.6)
Cardiac arrest	1 (0.6)	0	0	1 (0.6)	0
Hypertriglyceridemia	1 (0.6)	0	1 (0.6)	0	0
Hypokalemia	1 (0.6)	0	0	1 (0.6)	0
Neutropenia	1 (0.6)	0	0	1 (0.6)	0
Neutrophil count decreased	1 (0.6)	0	1 (0.6)	0	0
Noncardiac chest pain	1 (0.6)	0	1 (0.6)	0	0
Pleuritic pain	1 (0.6)	0	1 (0.6)	0	0
Proteinuria	1 (0.6)	0	1 (0.6)	0	0
Pulmonary arterial hypertension	1 (0.6)	0	1 (0.6)	0	0
Respiratory distress	1 (0.6)	0	0	0	1 (0.6)
Respiratory failure	1 (0.6)	0	0	1 (0.6)	0

*ALT* alanine aminotransferase, *AST* aspartate aminotransferase, *GGT* γ-glutamyl transferase

Of eight patients (4.8%) who discontinued avelumab because of a treatment-related AE, three (1.8%) discontinued because of an immune-related AE (autoimmune hepatitis [*n* = 2, both grade 3] and pemphigoid [*n* = 1, grade 2]); other treatment-related AEs resulting in discontinuation were elevated γ-glutamyl transferase (*n* = 2, grades 3 and 4), elevated aspartate aminotransferase (*n* = 1, grade 3), elevated creatine phosphokinase (*n* = 1, grade 1), and respiratory distress (*n* = 1, grade 5). Two patients (1.2%) had a treatment-related death, including the patient with acute liver failure described above and a patient with metastatic lesions of liver, lung, and soft tissues and a history of respiratory disorders (cough, dyspnea, and pneumonia) who died of respiratory distress. The patient with treatment-related respiratory distress also presented with unspecified sepsis, most likely secondary to healthcare-associated pneumonia, and extensive pulmonary metastasis.

### Antitumor activity

**Fig. 1:**
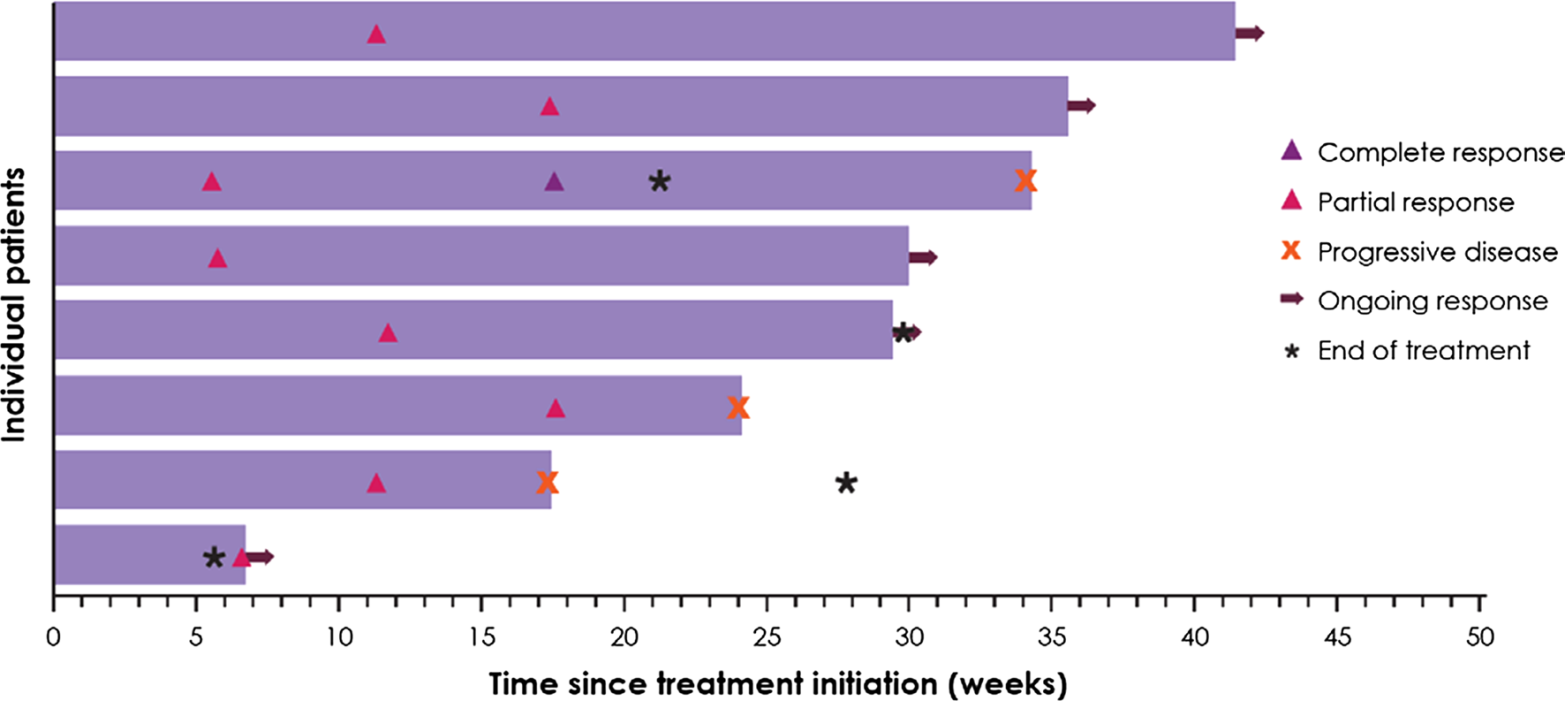
Time to and duration of response for patients with confirmed or unconfirmed responses

Based on investigator assessment, five patients had a confirmed objective response, including one complete response and four partial responses, resulting in a confirmed ORR of 3.0% (95% CI 1.0–6.8; Table 3). Notably, three of five confirmed responders (60.0%) had TNBC, resulting in an ORR of 5.2% in this subset (Appendix Table 9). The other two confirmed responders had HER2− ER/PR+ disease, and the ORR in this subset was 2.8% (Appendix Table 9). Four out of five responses were ongoing at data cutoff, and median duration of response was not reached (95% CI 28.7, ne). The unconfirmed ORR in the total population was 4.8%, including one complete response and seven partial responses, and the median time to response was 11.4 weeks (range 5.7–17.6 weeks) (Fig. 1). Stable disease was the best response in 42 patients (25.0%), and the disease control rate (DCR)—based on patients with a confirmed response or stable disease—was 28.0% (47 of 168) (Table 3). Within the TNBC subgroup, 15 patients (25.9%) had stable disease as best response, and the DCR was 31.0% (18 of 58). Of the 47 patients with a best response of complete response, partial response, or stable disease, 14 (29.8%) remained progression free for ≥ 24 weeks.

**Table 3:** Antitumor activity of avelumab

	Study population *N* = 168	TNBC subgroup *n* = 58
Complete response, *n* (%)	1 (0.6)	0
Partial response, *n* (%)	4 (2.4)	3 (5.2)
Stable disease,^a^ *n* (%)	42 (25.0)	15 (25.9)
Progressive disease, *n* (%)	106 (63.1)	38 (65.5)
Nonevaluable,^b^ *n* (%)	15 (8.9)	2 (3.4)
ORR (95% CI) (%)	3.0 (1.0 to 6.8)	5.2 (1.1 to 14.4)
DCR (%)	28.0	31.0
Median (95% CI) duration of response in confirmed responders (weeks)	ne (28.7 to ne)	ne (ne to ne)
Median (95% CI) progression-free survival (weeks)	5.9 (5.9 to 6.0)	5.9 (5.7 to 6.9)
Progression-free survival rate at 24 weeks (95% CI) (%)	10.1 (5.9 to15.5)	12.4 (5.2 to 22.8)
Median (95% CI) overall survival (months)	8.1 (6.4 to ne)	9.2 (4.3 to ne)
Overall survival rate at 12 months (95% CI) (%)	40.3 (29.6 to 50.7)	37.1 (18.3 to 56.2)

Treatment responses are based on confirmed response according per Response Criterion In Solid Tumors version 1.1

*DCR* disease control rate (defined as responses + stable disease), *ne* not estimable, *ORR* objective response rate

^a^Stable disease at the first post-baseline tumor assessment after 6 weeks was required to qualify for a best response of SD

^b^Includes “missing” and “not assessable”

**Fig. 2:**
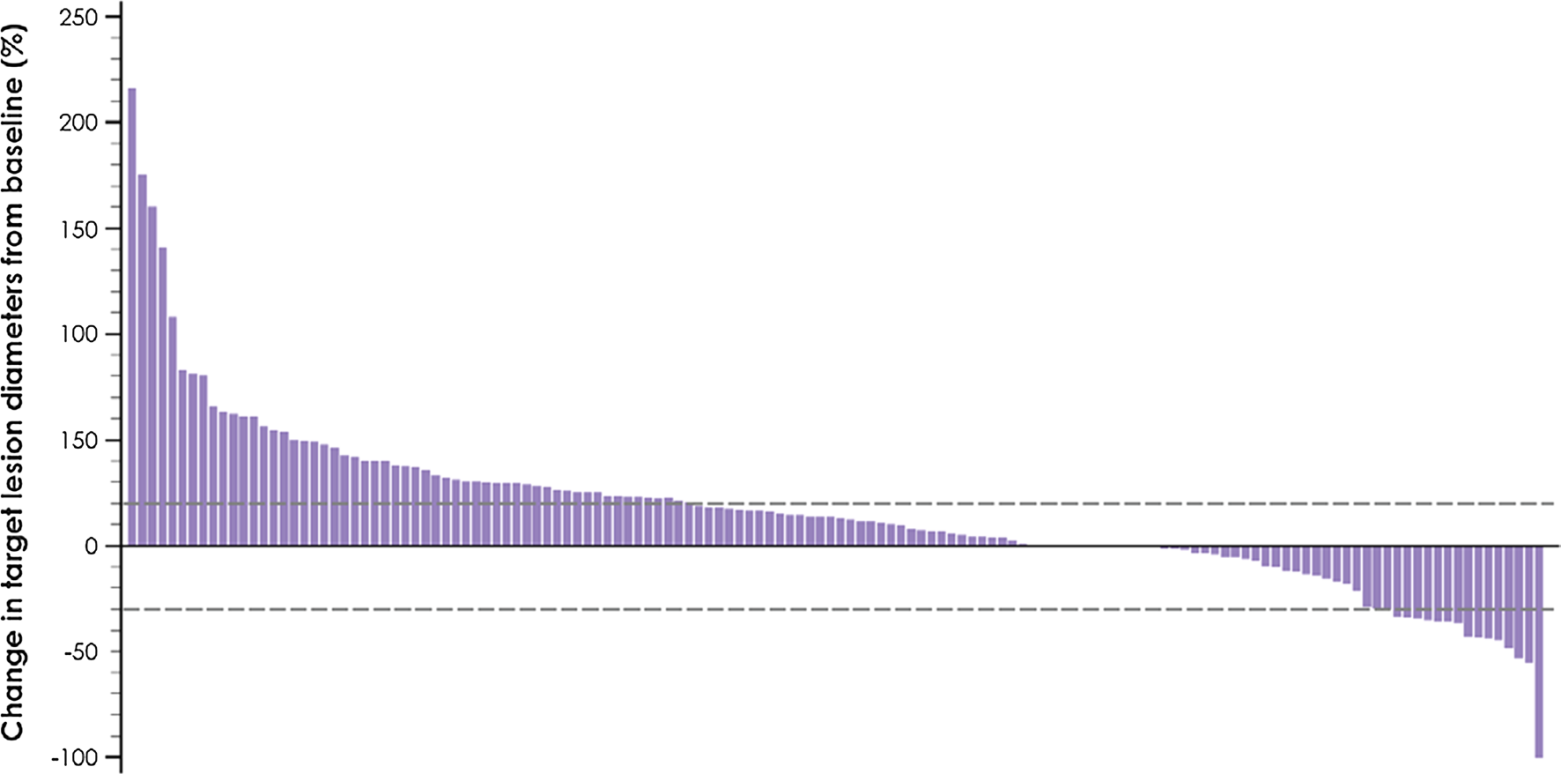
Best change in target lesions from baseline in 140 evaluable patients with baseline tumor assessment and ≥ 1 post-baseline assessment

**Fig. 3:**
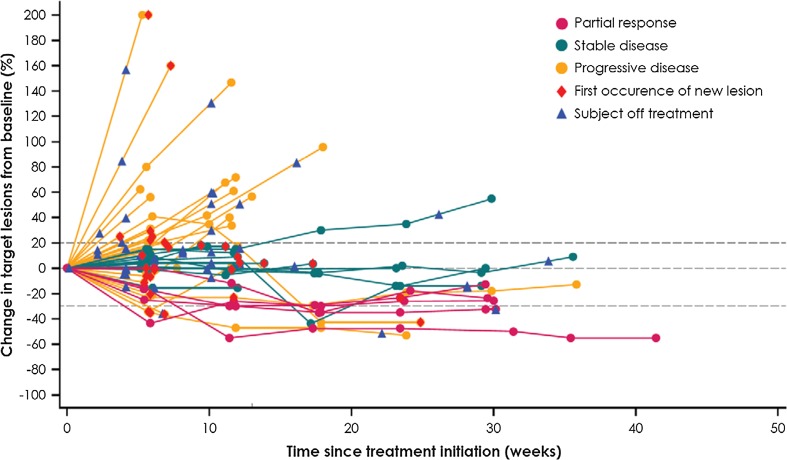
Percent change in target lesions from baseline in 46 evaluable patients with TNBC with baseline tumor assessment and ≥ 1 post-baseline assessment

Of 140 patients who had evaluable data for sum of target lesion diameter at baseline and on study, 39 patients (27.9%) experienced tumor shrinkage of any level. Sixteen (11.4%) of these patients had tumor shrinkage of ≥ 30%, including two patients with progressive disease by RECIST who had a partial response by modified immune-related response criteria (Fig. 2). Among 46 evaluable patients with TNBC, 21 (45.7%) had tumor shrinkage of any level, which was by ≥ 30% in ten patients (21.7%) (Fig. 3).

No trends for response were observed based on patient or disease characteristics, including age, race, ECOG status, and prior lines of therapy (Appendix Table 9). In addition, no efficacy trends were seen in subgroups defined by PD-L1 expression in tumor cells at different thresholds (Appendix Table 10). However, in evaluable patients with PD-L1+ or PD-L1− tumor-associated immune cells (10% staining cutoff), the ORR was 16.7% (2 of 12 patients) versus 1.6% (2 of 124 patients) in the overall group, and 22.2% (2 of 9 patients) versus 2.6% (1 of 39 patients) in patients with TNBC.

## Discussion

In this study of 168 heavily pretreated patients with MBC refractory to or progressing after standard-of-care therapy, avelumab monotherapy showed an acceptable safety profile with an incidence of grade ≥ 3 treatment-related AEs (13.7%) comparable with other anti-PD-L1/PD-1 therapies in MBC [17, 18]. Antitumor activity was modest, with a confirmed ORR of 3.0% based on one complete response and four partial responses. Of these five responders, three were in the TNBC subgroup (ORR 5.2%). Importantly, responses were durable, and the median duration of response was not reached during available follow-up. Tumor shrinkage occurred in 27.9% of evaluable patients in the overall MBC group and in 45.7% of patients with TNBC. The DCR was 28.0% in the total patient group and 31.0% in patients with TNBC. Preclinical studies suggest that avelumab may mediate tumor lysis through ADCC, indicating the presence of a potential second mechanism of action [21–23]. However, no clinical data are available to show that ADCC contributes to the clinical activity of avelumab. Importantly, the frequency of immune cell subsets is not decreased following treatment with avelumab [29].

To date, the use of PD-L1 as a predictive biomarker for MBC remains controversial given the use of different PD-L1 antibodies and detection assays, various PD-L1 expression cutoffs, and non-standardized test designs [30, 31]. Studies of pembrolizumab (anti-PD-1) and atezolizumab (anti-PD-L1) have included analyses of tumor PD-L1 expression in patients with TNBC [17, 19]. In the phase 1b KEYNOTE-012 study of pembrolizumab in PD-L1+ TNBC (*n* = 27 evaluable patients), PD-L1 positivity was defined as expression in the stroma or in ≥ 1% of tumor cells based on IHC staining using the 22C3 PD-L1 antibody. In this PD-L1+ cohort, the ORR was 18.5%, 37.5% of evaluable patients had tumor shrinkage of any level, and the DCR was 25.9% [17]. An exploratory analysis suggested an association between PD-L1 score (percentage of inflammatory and tumor cells staining for PD-L1) and the probability of response and progression-free survival with pembrolizumab. Similarly, in a phase 1a study of atezolizumab in 115 patients with heavily pretreated TNBC, of whom 71 were PD-L1+ (based on ≥ 5% of tumor-infiltrating immune cells [IHC score of 2/3 using the SP142 assay]) and were considered evaluable for efficacy, the unconfirmed ORR was 13% [19]. In our study, a possible trend toward a higher ORR was seen in patients with PD-L1+ versus PD-L1− tumor-associated immune cells, both in the overall population (16.7% vs. 1.6%) and in the TNBC subgroup (22.2% vs. 2.6%). The response rate to avelumab in patients with TNBC and PD-L1+ tumor-associated immune cells is comparable with the response rate of 18.5 and 13% reported for patients with PD-L1+ TNBC treated with pembrolizumab and atezolizumab, respectively [17, 19]. Although the small number of evaluable patients with PD-L1+ TILs in our study (*n* = 12) precludes any definitive conclusions, these data support the hypothesis that PD-L1 expression on TILs may predict response to checkpoint inhibitor therapy in breast cancer. In our study, unlike in the pembrolizumab and atezolizumab studies, PD-L1 expression was evaluated separately in tumor cells and tumor-associated immune cells as prespecified in the analysis plan, and the Dako PD-L1 IHC 73-10 pharmDx assay was used for the evaluation. Efforts to standardize testing for PD-L1 expression are underway, including the Blueprint PD-L1 IHC comparison project [32]. Data are not yet available to compare the performance of the 73-10 assay with that of other assays, although studies are ongoing, and the 73-10 assay will be included in phase 2 of the Blueprint study. Studies to characterize the potential of PD-L1 as a predictive biomarker for avelumab are ongoing.

In conclusion, our data show that the anti-PD-L1 antibody avelumab has a safety profile that is considered generally manageable and tolerable, and showed modest clinical activity in a heavily pretreated population of patients with MBC. Collectively, our findings and those of other studies suggest that durable clinical benefit can be achieved with anti-PD-1/PD-L1 monotherapy in a subset of patients with MBC, particularly TNBC [17–19]. Based on the results from single-agent immunotherapy in patients with MBC, studies of combination therapy that might increase the probability of treatment benefit are warranted, and promising clinical activity in TNBC has been reported for a treatment regimen of atezolizumab administered in combination with taxane chemotherapy (NCT01633970) and of pembrolizumab in combination with eribulin mesylate (NCT02513472) in preliminary studies [33, 34]. An ongoing phase 1b/2 study (JAVELIN Medley; NCT02554812), which includes a TNBC cohort, is currently assessing avelumab in combination with novel immunotherapies.

## Appendix

See Tables 4, 5, 6, 7, 8, 9, and 10.

**Table 4:** Additional patient demographics and disease characteristics

Characteristics, *n* (%)	*N* = 168
Prior surgery	161 (95.8)
Prior radiotherapy	139 (82.7)
Prior anticancer therapies in any setting	
1	5 (3.0)
2	27 (16.1)
≥ 3	136 (80.9)
Median (range)	4.0 (1–10)
Prior platinum regimen	
Carboplatin based	36 (21.4)
Cisplatin based	9 (5.4)
Type of prior anticancer therapy	
Chemotherapy	168 (100.0)
Hormonal therapy	103 (61.3)
Antibody therapy	30 (17.9)
Kinase inhibitor	19 (11.3)
Vaccines	1 (0.6)
Other	36 (21.4)
Intent of any prior therapy	
Neoadjuvant	64 (38.1)
Adjuvant	111 (66.1)
Metastatic	137 (81.5)
Locally advanced	30 (17.9)
Palliative	7 (4.2)
Best response to any prior anticancer therapy	
Complete response	3 (1.8)
Partial response	9 (5.4)
Stable disease	33 (9.6)
Progressive disease	94 (56.0)
Not assessable	1 (0.6)
Unknown	13 (7.7)
Not applicable	15 (8.9)

**Table 5:** Key eligibility criteria

Inclusion criteria	Exclusion criteria
Age ≥ 18 years	Prior therapy with any drug targeting T cell coregulatory proteins
Histologically confirmed MBC that is refractory to or progressive after standard-of-care therapy	Concurrent anticancer therapy within 4 weeks of start of trial treatment, use of hormonal agents within 7 days of start of trial treatment, or any other concurrent investigational treatment
No more than three prior lines of cytotoxic therapy for metastatic disease	Prior treatment with immunosuppressive agents such as steroids
Prior treatment with a taxane and an anthracycline, unless contraindicated	Major surgery ≤ 4 weeks prior to enrollment
Availability of a formalin-fixed, paraffin-embedded block containing tumor tissue or unstained tumor slides suitable for PD-L1 expression assessment	Previous malignant disease other than MBC within the last 5 years except for basal or squamous cell carcinoma of the skin or cervical carcinoma in situ
ECOG performance status score of 0 or 1	Metastases of the central nervous system
Measurable disease by RECIST version 1.1 or objective evidence of disease without a measurable lesion	Clinically significant illness, including infection, autoimmune disease (other than diabetes mellitus type 1, vitiligo, psoriasis, hypothyroid disease, or hyperthyroid disease not requiring immunosuppressive treatment), cardiovascular disease, or a psychiatric condition affecting the understanding or rendering of informed consent
Estimated life expectancy of ≥ 3 months	Persisting toxicity of grade > 1 related to prior therapy (except grade ≤ 2 sensory neuropathy)
Adequate renal, hepatic, and hematologic function	Known severe hypersensitivity to monoclonal antibodies, history of anaphylaxis, or uncontrolled asthma
Use of highly effective contraception	Vaccination (other than inactivated vaccines) within 55 days of the first dose of avelumab
Signed written informed consent	Pregnancy or lactation
	Known alcohol or drug abuse
	Legal incapacity or limited legal capacity

*ECOG* Eastern Cooperative Oncology Group, *MBC* metastatic breast cancer, *PD*-*L1* programmed death-ligand 1, *RECIST* Response Evaluation Criteria In Solid Tumors

**Table 6:** Prior cytotoxic therapies

Therapy	Patients (*N* = 168)
Cyclophosphamide	132 (78.6)
Paclitaxel	108 (64.3)
Doxorubicin	94 (56.0)
Docetaxel	86 (51.2)
Capecitabine	79 (47.0)
Fluorouracil	38 (22.6)
Carboplatin	36 (21.4)
Epirubicin	34 (20.2)
Trastuzumab	30 (17.9)
Bevacizumab	29 (17.3)
Everolimus	26 (15.5)
Nab-paclitaxel	24 (14.3)
Gemcitabine	19 (11.3)
Gemcitabine hydrochloride	17 (10.1)
Eribulin	16 (9.5)
Pegylated liposomal doxorubicin hydrochloride	16 (9.5)
Methotrexate	12 (7.1)
Vinorelbine tartrate	10 (6.0)
Cyclophosphamide with doxorubicin	9 (5.4)
Lapatinib	7 (4.2)
Pertuzumab	7 (4.2)
Cisplatin	6 (3.6)
Trastuzumab emtansine	6 (3.6)
Vinorelbine	6 (3.6)
Eribulin mesylate	3 (1.8)
Ixabepilone	3 (1.8)
Cisplatin with docetaxel	2 (1.2)
Cyclophosphamide with docetaxel/doxorubicin	2 (1.2)
Cyclophosphamide with epirubicin hydrochloride/fluorouracil	2 (1.2)
Cyclophosphamide with fluorouracil/methotrexate	2 (1.2)
Doxorubicin hydrochloride	2 (1.2)
Onartuzumab	2 (1.2)
Veliparib	2 (1.2)
PI3 kinase inhibitor	2 (1.2)
Doxorubicin/cyclophosphamide and paclitaxel	1 (0.6)
Carboplatin with gemcitabine	1 (0.6)
Cetuximab	1 (0.6)
5-Fluorouracil, epirubicin, and cyclophosphamide	1 (0.6)
Cyclophosphamide with epirubicin/fluorouracil	1 (0.6)
Epirubicin hydrochloride	1 (0.6)
Idarubicin	1 (0.6)
Irinotecan	1 (0.6)
Lapatinib ditosylate monohydrate	1 (0.6)
Neratinib	1 (0.6)
Olaparib	1 (0.6)
Panitumumab	1 (0.6)
Ruxolitinib	1 (0.6)
Sorafenib	1 (0.6)

**Table 7:** Adverse events (related or unrelated) of any grade in > 5% of patients or of grade ≥ 3 in any patient

Adverse event, *n* (%)	Patients (*N* = 168)					
	Any grade	Grade 1	Grade 2	Grade 3	Grade 4	Grade 5
Any event, *n* (%)	161 (95.8)	24 (14.3)	57 (33.9)	48 (28.6)	10 (6.0)	22 (13.1)
Fatigue	63 (37.5)	30 (17.9)	30 (17.9)	3 (1.8)	0	0
Nausea	49 (29.2)	29 (17.3)	19 (11.3)	1 (0.6)	0	0
Constipation	29 (17.3)	21 (12.5)	8 (4.8)	0	0	0
Decreased appetite	29 (17.3)	19 (11.3)	10 (6.0)	0	0	0
Diarrhea	29 (17.3)	22 (13.1)	7 (4.2)	0	0	0
Vomiting	25 (29.2)	14 (8.3)	9 (5.4)	2 (1.2)	0	0
Back pain	24 (14.3)	10 (6.0)	7 (4.2)	7 (4.2)	0	0
Cough	24 (14.3)	19 (11.3)	5 (3.0)	0	0	0
Dyspnea	24 (14.3)	10 (6.0)	4 (2.4)	9 (5.4)	1 (0.6)	0
Infusion-related reaction	24 (14.3)	6 (3.6)	18 (10.7)	0	0	0
Arthralgia	23 (13.7)	15 (8.9)	3 (1.8)	5 (3.0)	0	0
Dyspnea exertional	23 (13.7)	15 (8.9)	6 (3.6)	1 (0.6)	1 (0.6)	0
Pyrexia	21 (12.5)	18 (10.7)	2 (1.2)	1 (0.6)	0	0
Abdominal pain	19 (11.3)	11 (6.5)	5 (3.0)	3 (1.8)	0	0
Abdominal pain upper	19 (11.3)	7 (4.2)	11 (6.5)	1 (0.6)	0	0
Anemia	17 (10.1)	2 (1.2)	5 (3.0)	9 (5.4)	1 (0.6)	0
Pleural effusion	17 (10.1)	4 (2.4)	4 (2.4)	8 (4.8)	1 (0.6)	0
Urinary tract infection	17 (10.1)	5 (3.0)	12 (7.1)	0	0	0
Asthenia	16 (9.5)	7 (4.2)	6 (3.6)	3 (1.8)	0	0
Edema peripheral	15 (8.9)	8 (4.8)	6 (3.6)	1 (0.6)	0	0
Headache	15 (8.9)	13 (7.7)	2 (1.2)	0	0	0
Elevated AST	14 (8.3)	2 (1.2)	6 (3.6)	6 (3.6)	0	0
Influenza-like illness	14 (8.3)	13 (7.7)	1 (0.6)	0	0	0
Pain in extremity	13 (7.7)	5 (3.0)	5 (3.0)	3 (1.8)	0	0
Disease progression	12 (7.1)	0	0	1 (0.6)	1 (0.6)	10 (6.0)
Musculoskeletal pain	12 (7.1)	4 (2.4)	8 (4.8)	0	0	0
Noncardiac chest pain	12 (7.1)	4 (2.4)	3 (1.8)	5 (3.0)	0	0
Abdominal distension	11 (6.5)	6 (3.6)	4 (2.4)	1 (0.6)	0	0
Anxiety	11 (6.5)	6 (3.6)	5 (3.0)	0	0	0
Decreased weight	11 (6.5)	7 (4.2)	4 (2.4)	0	0	0
Dizziness	11 (6.5)	7 (4.2)	3 (1.8)	1 (0.6)	0	0
Elevated ALT	11 (6.5)	6 (3.6)	4 (2.4)	1 (0.6)	0	0
Hypertension	11 (6.5)	4 (2.4)	5 (3.0)	2 (1.2)	0	0
Insomnia	11 (6.5)	6 (3.6)	5 (3.0)	0	0	0
Musculoskeletal chest pain	11 (6.5)	7 (4.2)	3 (1.8)	1 (0.6)	0	0
Hypothyroidism	10 (6.0)	2 (1.2)	8 (4.8)	0	0	0
Muscle spasms	10 (6.0)	10 (6.0)	0	0	0	0
Neck pain	10 (6.0)	4 (2.4)	6 (3.6)	0	0	0
Dry skin	9 (5.4)	8 (4.8)	1 (0.6)	0	0	0
Hypokalemia	9 (5.4)	3 (1.8)	5 (3.0)	0	1 (0.6)	0
Pruritus	9 (5.4)	7 (4.2)	2 (1.2)	0	0	0
Chest pain	8 (4.8)	5 (3.0)	2 (1.2)	1 (0.6)	0	0
Elevated blood alkaline phosphatase	7 (4.2)	2 (1.2)	3 (1.8)	2 (1.2)	0	0
Myalgia	7 (4.2)	6 (3.6)	0	1 (0.6)	0	0
Elevated GGT	6 (3.6)	1 (0.6)	1 (0.6)	2 (1.2)	2 (1.2)	0
Flank pain	5 (3.0)	2 (1.2)	2 (1.2)	1 (0.6)	0	0
Hyponatremia	5 (3.0)	2 (1.2)	1 (0.6)	2 (1.2)	0	0
Hypoxia	5 (3.0)	0	3 (1.8)	2 (1.2)	0	0
Pneumonia	5 (3.0)	0	1 (0.6)	4 (2.4)	0	0
Hypercalcemia	4 (2.4)	2 (1.2)	1 (0.6)	0	1 (0.6)	0
Thrombocytopenia	4 (2.4)	1 (0.6)	0	0	3 (1.8)	0
Respiratory failure	4 (2.4)	0	0	0	2 (1.2)	2 (1.2)
Autoimmune hepatitis	3 (1.8)	0	0	3 (1.8)	0	0
Axillary pain	3 (1.8)	1 (0.6)	1 (0.6)	1 (0.6)	0	0
General physical health deterioration	3 (1.8)	0	0	2 (1.2)	0	1 (0.6)
Hyperglycemia	3 (1.8)	0	2 (1.2)	1 (0.6)	0	0
Lymphocyte count decreased	3 (1.8)	0	2 (1.2)	1 (0.6)	0	0
Metastatic pain	3 (1.8)	1 (0.6)	0	2 (1.2)	0	0
Pleuritic pain	3 (1.8)	1 (0.6)	1 (0.6)	1 (0.6)	0	0
Pneumonitis	3 (1.8)	1 (0.6)	1 (0.6)	1 (0.6)	0	0
Pneumothorax	3 (1.8)	1 (0.6)	1 (0.6)	1 (0.6)	0	0
Proteinuria	3 (1.8)	2 (1.2)	0	1 (0.6)	0	0
Sinus tachycardia	3 (1.8)	1 (0.6)	1 (0.6)	1 (0.6)	0	0
Atrial fibrillation	2 (1.2)	0	0	1 (0.6)	1 (0.6)	0
Cardiac tamponade	2 (1.2)	0	0	1 (0.6)	1 (0.6)	0
Deep vein thrombosis	2 (1.2)	0	1 (0.6)	1 (0.6)	0	0
Hematuria	2 (1.2)	1 (0.6)	0	1 (0.6)	0	0
Hepatomegaly	2 (1.2)	1 (0.6)	0	1 (0.6)	0	0
Hypophosphatemia	2 (1.2)	0	0	2 (1.2)	0	0
International normalized ratio increased	2 (1.2)	1 (0.6)	0	1 (0.6)	0	0
Jugular vein thrombosis	2 (1.2)	0	0	2 (1.2)	0	0
Metastases to meninges	2 (1.2)	0	1 (0.6)	0	1 (0.6)	0
Neutrophil count decreased	2 (1.2)	1 (0.6)	0	1 (0.6)	0	0
Platelet count decreased	2 (1.2)	0	1 (0.6)	1 (0.6)	0	0
Radiation pneumonitis	2 (1.2)	1 (0.6)	0	1 (0.6)	0	0
Rash papular	2 (1.2)	1 (0.6)	0	1 (0.6)	0	0
Respiratory distress	2 (1.2)	0	0	1 (0.6)	0	1 (0.6)
Acute hepatic failure	1 (0.6)	0	0	0	0	1 (0.6)
Acute kidney injury	1 (0.6)	0	0	1 (0.6)	0	0
Ataxia	1 (0.6)	0	0	1 (0.6)	0	0
Bile duct obstruction	1 (0.6)	0	0	1 (0.6)	0	0
Brain injury	1 (0.6)	0	0	0	0	1 (0.6)
Bronchial obstruction	1 (0.6)	0	0	1 (0.6)	0	0
Burning sensation	1 (0.6)	0	0	1 (0.6)	0	0
Cardiac arrest	1 (0.6)	0	0	0	1 (0.6)	0
Cardiac failure	1 (0.6)	0	0	0	1 (0.6)	0
Cardio-respiratory arrest	1 (0.6)	0	0	0	0	1 (0.6)
Cellulitis	1 (0.6)	0	0	1 (0.6)	0	0
Cholestasis	1 (0.6)	0	0	1 (0.6)	0	0
Coccydynia	1 (0.6)	0	0	1 (0.6)	0	0
Dental caries	1 (0.6)	0	0	1 (0.6)	0	0
Electrocardiogram QT prolonged	1 (0.6)	0	0	1 (0.6)	0	0
Elevated blood potassium	1 (0.6)	0	0	1 (0.6)	0	0
Elevated hepatic enzyme	1 (0.6)	0	0	1 (0.6)	0	0
Failure to thrive	1 (0.6)	0	0	1 (0.6)	0	0
Hemoptysis	1 (0.6)	0	0	1 (0.6)	0	0
Hepatic encephalopathy	1 (0.6)	0	0	0	1 (0.6)	0
Hepatic failure	1 (0.6)	0	0	0	0	1 (0.6)
Hyperbilirubinemia	1 (0.6)	0	0	1 (0.6)	0	0
Hypercalcemia of malignancy	1 (0.6)	0	0	0	1 (0.6)	0
Hypertriglyceridemia	1 (0.6)	0	0	1 (0.6)	0	0
Infection	1 (0.6)	0	0	1 (0.6)	0	0
Liver disorder	1 (0.6)	0	0	1 (0.6)	0	0
Malignant neoplasm progression	1 (0.6)	0	0	0	0	1 (0.6)
Malignant pleural effusion	1 (0.6)	0	0	1 (0.6)	0	0
Malnutrition	1 (0.6)	0	0	1 (0.6)	0	0
Metastases to peripheral nervous system	1 (0.6)	0	0	0	1 (0.6)	0
Monoparesis	1 (0.6)	0	0	1 (0.6)	0	0
Myelodysplastic syndrome	1 (0.6)	0	0	0	1 (0.6)	0
Neutropenia	1 (0.6)	0	0	0	1 (0.6)	0
Obstructive uropathy	1 (0.6)	0	0	1 (0.6)	0	0
Pain in hip, back, and right lower leg	1 (0.6)	0	0	1 (0.6)	0	0
Paraparesis	1 (0.6)	0	0	0	1 (0.6)	0
Pathological fracture	1 (0.6)	0	0	1 (0.6)	0	0
Pleural fistula	1 (0.6)	0	0	1 (0.6)	0	0
Pulmonary arterial hypertension	1 (0.6)	0	0	1 (0.6)	0	0
Pulmonary embolism	1 (0.6)	0	0	0	0	1 (0.6)
Pulmonary hypertension	1 (0.6)	0	0	0	0	1 (0.6)
Sepsis	1 (0.6)	0	0	0	0	1 (0.6)
Spinal cord compression	1 (0.6)	0	0	1 (0.6)	0	0
Tumor invasion	1 (0.6)	0	0	0	0	1 (0.6)
Tumor pain	1 (0.6)	0	0	1 (0.6)	0	0

*ALT* alanine aminotransferase, *AST* aspartate aminotransferase, *GGT* γ-glutamyl transferase

**Table 8:** Potential immune-related, treatment-related adverse events by grade

Adverse event, *n* (%)	Patients (*N* = 168)					
	Any grade	Grade 1	Grade 2	Grade 3	Grade 4	Grade 5
Any event, *n* (%)	**17 (10.1)**	**4 (2.4)**	**9 (5.4)**	**3 (1.8)**	**1 (0.6)**	**0**
Hypothyroidism	8 (4.8)	1 (0.6)	7 (4.2)	0	0	0
Autoimmune hepatitis^a^	3 (1.8)	0	0	3 (1.8)	0	0
Pneumonitis	3 (1.8)	1 (0.6)	1 (0.6)	1 (0.6)	0	0
Thrombocytopenia	2 (1.2)	1 (0.6)	0	0	1 (0.6)	0
ANA positive	1 (0.6)	1 (0.6)	0	0	0	0
Dry eye	1 (0.6)	1 (0.6)	0	0	0	0
Elevated rheumatoid factor	1 (0.6)	1 (0.6)	0	0	0	0
Hyperthyroidism	1 (0.6)	1 (0.6)	0	0	0	0
Pemphigoid^b^	1 (0.6)	0	1 (0.6)	0	0	0

The numbers in bold are the total number of immune related evens according to grade

*ANA* antinuclear antibody

^a^Autoimmune hepatitis temporarily resolved with steroid treatment, but led to discontinuation in two patients; the third patient who experienced autoimmune hepatitis died of acute liver failure in a setting of progressive liver metastasis

^b^Pemphigoid resolved following drug interruption but ultimately led to treatment discontinuation

**Table 9:** Confirmed ORR by subgroup

Subgroup	*n*/*N*1	ORR % (95% CI)
Age (years)		
< 65	4/140	2.9 (0.8, 7.2)
≥ 65	1/28	3.6 (0.1, 18.3)
Race		
White	3/143	2.1 (0.4, 6.0)
Black	2/25	8.0 (1.0, 26.0)
ECOG		
0	3/83	3.6 (0.8, 10.2)
≥ 1	2/85	2.4 (0.3, 8.2)
Prior lines for metastatic disease^a^		
≤ 1	2/45	4.4 (0.5, 15.1)
2	2/35	5.7 (0.7,19.2)
≥ 3	1/88	1.1 (0, 6.2)
Biomarker group		
TNBC	3/58	5.2 (1.1, 14.4)
HER2− (ER+ or PR+)	2/72	2.8 (0.3, 9.7)
HER2+	0/26	0 (0, 13.2)
Unknown^b^	0/12	0 (0, 26.5)

*ECOG PS* Eastern Cooperative Oncology Group performance status, *ER* estrogen receptor, *HER2* human epidermal growth factor receptor 2, *N1* number of evaluable patients, *ORR* objective response rate, *PR* progesterone receptor, *TNBC* triple-negative breast cancer, *N1* number of evaluable patients

^a^Regimen for locally advanced/metastatic disease may have included hormonal therapy, either alone or in combination with chemotherapy. Systemic therapies that were not necessarily cytotoxic are included in the number of prior regimens reported here, but the number of cytotoxic therapies permitted was ≤ 3

^b^ Unknown molecular subtype was due to incomplete information in the medical records database (ER/PR status known, but HER2 status unknown in four patients) or to information collected retrospectively (molecular subtype status was from post-baseline samples in eight patients and, therefore, was not used for baseline characterization

**Table 10:** Confirmed response, progression-free survival, and overall survival in patient subgroups defined by PD-L1 expression in tumor or immune cells

	PD-L1+	PD-L1−	*P* value^a^	Hazard ratio (95% CI)
≥ 1% Tumor cells, any intensity				
Prevalence, *n*	85	51	–	–
ORR (95% CI), *n*/*N*1 (%)	2/85 (2.4 [0.3, 8.2])	2/51 (3.9 [0.5, 13.5])	0.631	–
Median PFS (95% CI) (weeks)	5.9 (5.7, 6.0)	6.0 (5.9, 6.0)	–	1.183 (0.815, 1.716)
PFS rate at 24 weeks (95% CI) (%)	6.2 (2.1, 13.6)	12.8 (5.3, 23.8)	–	
Median OS (95% CI) (months)	6.5 (3.7, 9.2)	8.3 (6.3, ne)	–	1.331 (0.815, 2.174)
OS rate at 12 months (95% CI) (%)	35.6 (24.2,47.1)	36.8 (14.8, 59.3)	–	
≥ 5% Tumor cells, any intensity				
Prevalence, *n*	23	113	–	–
ORR (95% CI), *n*/*N*1 (%)	1/23 (4.3 [0.1, 21.9])	3/113 (2.7 [0.6, 7.6])	0.528	–
Median PFS (95% CI) (weeks)	6.0 (5.7, 7.1)	5.9 (5.9, 6.0)	–	0.782 (0.473, 1.290)
PFS rate at 24 weeks (95% CI) (%)	15.5 (3.3, 36.0)	7.7 (3.6, 13.8)	–	
Median OS (95% CI) (months)	6.5 (2.2, ne)	7.5 (5.1, 11.3)	–	1.057 (0.556, 2.010)
OS rate at 12 months (95% CI) (%)	ne	35.9 (23.7, 48.2)	–	
≥ 25% Tumor cells, high intensity (≥ 2+)				
Prevalence, *n*	3	133	–	–
ORR (95% CI), *n*/*N*1 (%)	0/3 (0 [0, 70.8])	4/133 (3.0 [0.8,7.5])	1.000	–
Median PFS (95% CI) (weeks)	6.0 (5.4, ne)	5.9 (5.9, 6.0)	–	0.695 (0.172, 2.813)
PFS rate at 24 weeks (95% CI) (%)	ne	8.6 (4.5, 14.5)	–	
Median OS (95% CI) (months)	9.2 (ne, ne)	6.8 (4.9, 10.8)	–	0.441 (0.061, 3.177)
OS rate at 12 months (95% CI) (%)	0 (ne, ne)	36.3 (25.0, 47.6)	–	
≥ 10% Tumor-associated immune cells, any intensity				
Prevalence, *n*	12	124	–	–
ORR (95% CI), *n*/*N*1 (%)	2/12 (16.7 [2.1, 48.4])	2/124 (1.6 [0.2, 5.7])	0.039	–
Median PFS (95% CI) (weeks)	6.1 (2.3, 24,1)	5.9 (5.9, 6.0)	–	0.656 (0.341, 1.263)
PFS rate at 24 weeks (95% CI) (%)	25.0 (6.0, 50.5)	7.0 (3.2, 12.9)	–	
Median OS (95% CI) (months)	11.3 (1.4, ne)	6.8 (4.7, 9.2)	–	0.620 (0.250, 1.541)
OS rate at 12 months (95% CI) (%)	33.3 (1.7, 74.4)	37.4 (27.4, 47.4)	–	

*ne* not estimable, *N1* number of patients with evaluable PD-L1 expression, *ORR* objective response rate, *OS* overall survival, *PD*-*L1* programmed death-ligand 1, *PFS* progression-free survival

^a^Fisher’s exact test
